# Free-breathing 3D phase-resolved functional lung MRI vs breath-hold hyperpolarized ^129^Xe ventilation MRI in patients with chronic obstructive pulmonary disease and healthy volunteers

**DOI:** 10.1007/s00330-024-10893-3

**Published:** 2024-07-26

**Authors:** Filip Klimeš, Agilo Luitger Kern, Andreas Voskrebenzev, Marcel Gutberlet, Robert Grimm, Robin Aaron Müller, Lea Behrendt, Till Frederik Kaireit, Julian Glandorf, Tawfik Moher Alsady, Frank Wacker, Jens M. Hohlfeld, Jens Vogel-Claussen

**Affiliations:** 1 Institute of Diagnostic and Interventional Radiology, Hannover Medical School, Hanover, Germany; 2 Biomedical Research in Endstage and Obstructive Lung Disease Hannover (BREATH), Member of the German Centre for Lung Research, Hanover, Germany; 3 MR Application Predevelopment, Siemens Healthineers AG, Erlangen, Germany; 4 Department of Clinical Airway Research, Fraunhofer Institute for Toxicology and Experimental Medicine, Hanover, Germany; 5 Department of Respiratory Medicine, Hannover Medical School, Hanover, Germany

**Keywords:** Chronic obstructive pulmonary disease, Magnetic resonance imaging, Ventilation, Lung

## Abstract

**Objectives:**

3D phase-resolved functional lung (PREFUL) MRI offers evaluation of pulmonary ventilation without inhalation of contrast agent. This study seeks to compare ventilation maps obtained from 3D PREFUL MRI with a direct ventilation measurement derived from ^129^Xe MRI in both patients with chronic obstructive pulmonary disease (COPD) and healthy volunteers.

**Methods:**

Thirty-one patients with COPD and 12 healthy controls underwent free-breathing 3D PREFUL MRI and breath-hold ^129^Xe MRI at 1.5 T. For both MRI techniques, ventilation defect (VD) maps were determined and respective ventilation defect percentage (VDP) values were computed. All parameters of both techniques were compared by Spearman correlation coefficient (*r*) and the differences between VDP values were quantified by Bland–Altman analysis and tested for significance using Wilcoxon signed-rank test. In a regional comparison of VD maps, spatial overlap and Sørensen–Dice coefficients of healthy and defect areas were computed.

**Results:**

On a global level, all 3D PREFUL VDP values correlated significantly to VDP measure derived by ^129^Xe ventilation imaging (all *r* > 0.65; all *p* < 0.0001). ^129^Xe VDP was significantly greater than 3D PREFUL derived VDP_RVent_ (mean bias = 10.5%, *p* < 0.001) and VDP_FVL-CM_ (mean bias = 11.3%, *p* < 0.0001) but not for VDP_Combined_ (mean bias = 1.7%, *p* = 0.70). The total regional agreement of ^129^Xe and 3D PREFUL VD maps ranged between 60% and 63%.

**Conclusions:**

Free-breathing 3D PREFUL MRI showed a strong correlation with breath-hold hyperpolarized ^129^Xe MRI regarding the VDP values and modest differences in the detection of VDs on a regional level.

**Clinical relevance statement:**

3D PREFUL MRI correlated with ^129^Xe MRI, unveiling regional differences in COPD defect identification. This proposes 3D PREFUL MRI as a ventilation mapping surrogate, eliminating the need for extra hardware or inhaled gases.

**Key Points:**

- *Current non-invasive evaluation techniques for lung diseases have drawbacks;* ^*129*^*Xe MRI is limited by cost and availability*.
- *3D PREFUL MRI correlated with* ^*129*^*Xe MRI, with regional differences in identifying COPD defects*.
- *3D PREFUL MRI can provide ventilation mapping without the need for additional hardware or inhaled gases*.

## Introduction

Pulmonary diseases such as chronic obstructive lung disease (COPD) pose a significant burden on global healthcare systems [1]. While pulmonary function tests serve as the current non-invasive clinical diagnostic method, they lack sensitivity to early signs of pulmonary diseases and spatial information. Computed tomography (CT) is considered the gold standard for structural thoracic imaging [2], while single photon emission computed tomography (SPECT) is routinely used for functional assessment [3]. However, SPECT has limitations such as low spatial resolution and prolonged exam times. Additionally, both CT and SPECT involve ionizing radiation, limiting their utility in therapy monitoring, especially in vulnerable patient groups like young children or pregnant women.

These limitations have led to the development of magnetic resonance imaging (MRI) methods. Despite being radiation-free and suitable for therapy monitoring, MRI’s clinical routine use is hindered by pulmonary tissue properties [4], particularly the low tissue density of the lung and weakened measurable signal at the air–tissue interfaces. Hyperpolarized ^129^Xe MRI addresses these challenges by directly visualizing inhaled gas distribution with high spatial resolution across all imaging planes [5]. This technique has demonstrated sensitivity to changes in lung microstructure and impaired lung function across a diverse range of pulmonary conditions, including cystic fibrosis [6], asthma [7], COPD [8, 9], and idiopathic pulmonary fibrosis [10]. While hyperpolarized ^129^Xe MRI has been shown to be well repeatable [11, 12], its limited availability and requirement for additional expensive equipment pose challenges.

In the past two decades, proton-based methods [13–15] have gained interest due to their patient-friendly free breathing acquisitions and cost-effectiveness. The phase-resolved functional lung (PREFUL) MRI, developed using the clinically established 2D spoiled gradient echo sequence, assesses the whole respiratory and cardiac cycles [16]. The PREFUL technique has been validated by comparison to established techniques [17, 18], tested for reproducibility and repeatability [11, 19], and has shown responsiveness to dual bronchodilator therapy in COPD [20]. Further advancements include the introduction of 3D techniques [21–23] to match the spatial resolution of ^129^Xe MRI, facilitating the assessment of pulmonary ventilation throughout the entire lung volume. Unlike previous comparisons of 2D proton-based methods with established techniques [11, 24–26], quantitative comparison between ventilation defect percentage (VDP) [27, 28] values obtained using 3D proton-based MRI methods and direct measurement of pulmonary ventilation using ^129^Xe MRI is lacking.

This study seeks to conduct a regional comparison of ventilation maps generated using free-breathing 3D PREFUL MRI with breath-hold hyperpolarized ^129^Xe MRI in patients with COPD and healthy controls.

## Materials and methods

The institutional review board approved the study, and written informed consent was obtained from all study participants. COPD patients were recruited under approval number 07423, while healthy volunteers were recruited under approval number 10020.

### Subject participants

Thirty-one COPD patients (17 males, age range: 47–73 years), and twelve healthy, non-smoking controls (7 males, age range: 24–72 years) underwent MR imaging on a 1.5T scanner (MAGNETOM Avanto, Siemens Healthineers). All subjects had common exclusion criteria, including (a) inability to undergo MR imaging (e.g., implanted pacemaker, cochlear implants or claustrophobia); (b) additional risks (e.g., pregnancy or chest surgery); and (c) age under 18 years.

### Spirometry

The spirometry was performed in accordance with the current guidelines of the American Thoracic Society [29] to determine forced expiratory volume in 1 s (FEV_1_), forced vital capacity (FVC), and FEV_1_/FVC ratio.

### MR protocol

#### 3D PREFUL MRI

3D PREFUL MRI datasets have been acquired using a stack-of-stars 3D gradient echo research pulse sequence with a golden-angle increment, lasting 7.3–8.3 min depending on the required number of partitions to cover the entire lung [23]. 3D PREFUL acquisition utilized a 6-channel body phased array coil in conjunction with a 12-channel spine matrix coil. Details on MR sequence parameters of the 3D PREFUL acquisition are listed in Supporting Table S1. The reconstruction of respiratory-resolved images with a spatial resolution of 3.9 mm isotropic is described in the Supporting Information and the overview of the 3D PREFUL method is illustrated in Supporting Fig. 1.

#### ^129^Xe MRI

^129^Xe MRI was performed using a custom-made birdcage transmit and 16-channel receive coil (Rapid Biomedical, Rimpar, Germany) at the same MR scanner as 3D PREFUL MRI. For ventilation imaging, we employed a 3D gradient echo stack-of-stars sequence with standard linear ordering. The imaging parameters are detailed in Supporting Table S1. For anatomical reference, a ^1^H scan (echo time/repetition time = 0.99/3 ms, flip angle = 5°) with a spatial resolution of 2.6 mm (slice thickness of 15 mm) was performed using the MRI’s body coil in the same breathing position. For ^129^Xe MRI, study participants inhaled a mixture of hyperpolarized ^129^Xe gas (600–800 mL) and nitrogen. To ensure the same breathing position between ^129^Xe ventilation and ^1^H scan, study participants inhaled a bag containing 1 L of air/xenon mixture on both occasions, starting from functional residual capacity.

### MR image analysis

The MATLAB software (version R2020b, MathWorks) was utilized for the image analysis of 3D PREFUL and ^129^Xe MRI ventilation parameters.

#### Ventilation parameters 3D PREFUL MRI

For image analysis, a convolutional neural network segmented the thorax cavity of each study participant’s end-inspiratory image [30]. Subsequently, lung vessels were identified and excluded from the thorax cavity mask [31], resulting in the final lung parenchyma mask for 3D PREFUL MRI. Manual correction of the final lung parenchyma segmentation, if necessary, was conducted by a scientist with 7 years of experience in pulmonary MR imaging, under the supervision of an experienced radiologist with 20 years of experience.

Afterward, ventilation defect (VD) maps were computed based on previously published thresholds [32] for:

- a static ventilation (RVent) parameter [33] that considers only two respiratory phases with maximal ventilation amplitude (VD threshold of 90 percentile multiplied by a factor of 0.4),
- and dynamic flow-volume-loop cross-correlation metric (FVL-CM) parameter [34] that assesses how well the ventilation dynamics of each voxel correlate with healthy reference ventilation dynamics (VD threshold of 0.9) [35].

To increase sensitivity for ventilation abnormalities depicted by 3D PREFUL MRI, both VD maps were combined using the logical OR operation, which resulted in the VD_Combined_ map. All three VD maps, as well as their respective VDP values, were considered for comparison to ^129^Xe imaging.

#### Ventilation parameters ^129^Xe MRI

The ^129^Xe ventilation image was reconstructed using the Berkeley advanced reconstruction toolbox [36]. Bias fields from receiver coil sensitivity variation and transmit field inhomogeneity were compensated for using N4 bias field correction [37]. Quantification of VDP_Xe_ was conducted by applying a region-growing algorithm to generate a mask of the thoracic cavity in the ^1^H gradient echo image, followed by the application of a linear binning [38] algorithm on ^129^Xe ventilation images.

#### Slice thickness alignment and coregistration of 3D PREFUL MRI and ^129^Xe MRI

^129^Xe MRI images were considered as a gold standard ventilation measurement in this comparison. To ensure voxel-wise comparability between 3D PREFUL MRI and ^129^Xe MRI within the same study participant, alignment was essential. This involved matching the slice thickness of the respiratory phase resolved morphological images (3.9 mm) from 3D PREFUL MRI to those morphological images acquired prior to the ^129^Xe ventilation MRI (15 mm) using the same breathing maneuver. Additionally, coregistration of 3D PREFUL MRI images to ^129^Xe MRI morphological images was performed using the Advanced normalization tool registration toolbox [39]. Finally, 3D PREFUL VD maps were reformatted and coregistered to ^129^Xe MRI using the obtained transformation fields to ensure spatial correspondence of all voxels.

### Statistical analysis

Statistical analysis utilized MATLAB (R2020b, MathWorks) and JMP Pro 16 (SAS Institute). Data normality was assessed using the Shapiro–Wilk test. Nonparametric statistic tests were chosen due to non-normal distributions (all *p* ≤ 0.0341), except body mass index (BMI) and FVC in % predicted value (both *p* ≥ 0.7870).

Demographics, spirometry, and MR imaging comparisons between COPD and healthy volunteers employed the Wilcoxon rank sum test, with significance set at *p* < 0.05.

The relationship between VDP values derived by 3D PREFUL MRI and ^129^Xe MRI was assessed by Spearman correlation, with the significance of 3D PREFUL VDP and ^129^Xe MRI VDP values evaluated using paired Wilcoxon signed rank test (Bonferroni adjusted significance level of 0.0167).

The regional analysis included calculating of the spatial overlap, defined as the percentage of voxels within the lung parenchyma mask identified as both healthy and exhibiting a VD by both methods. Further, the agreement of healthy and defect voxels in both 3D PREFUL and ^129^Xe MRI was quantified by the Sørensen–Dice coefficient.

VDP parameters of 3D PREFUL and ^129^Xe MRI were correlated to spirometry outcomes by Spearman correlation. The correlations were tested for significance with Bonferroni's corrected *p* value of 0.0125.

## Results

### Study participants

The demographic characteristics, spirometry, and MRI results of the study participants are provided in Table 1. Except for BMI, significant differences were observed in all demographic (age), spirometric (FEV_1_ in % pred., FVC in % pred. and FEV_1_/FVC ratio in % pred.), and VDP parameters (of both MRI techniques) between COPD and healthy volunteers (all *p* < 0.0205).

**Table 1:** Study participant's demographics, spirometry, and MRI (3D PREFUL and ^129^Xe)-derived ventilation parameters

		All study participants, (*n* = 43)	COPD patients, (*n* = 31)	Healthy volunteers, (*n* = 12)	*p* (COPD vs healthy)
Demography	Sex (% male)	56	55	58	–
	Age (years)	64 (54–68)	67 (60–70)	35 (28–61)	0.0009*
	BMI (kg/m^2^)	26 (22–29)	26 (24–29)	24 (21–26)	0.38
Spirometry	FEV_1_ (% pred.)	55.0 (39.1–81.5)	42.4 (36.7–56.5)	96.5 (85.3–102.3)	< 0.0001*
	FVC (% pred.)	90.6 (80.6–99.0)	86.4 (76.9–95.9)	95.0 (91.8–101.0)	0.0205*
	FEV_1_/FVC (% pred.)	58.4 (42.9–88.5)	52.1 (41.7–63.1)	95.5 (92.5–102.0)	< 0.0001*
	GOLD I, II, III, IV	–	0, 12, 14, 5	–	–
3D PREFUL	VDP_RVent_ (%)	17.7 (8.6–30.3)	27.8 (15.7–34.2)	3.7 (2.8–7.8)	< 0.0001*
	VDP_FVL-CM_ (%)	16.7 (7.0–33.8)	25.2 (14.3–35.0)	1.4 (0.4–3.4)	< 0.0001*
	VDP_Combined_ (%)	26.6 (11.1–45.9)	38.4 (21.4–48.2)	4.8 (3.3–9.1)	< 0.0001*
^129^Xe	VDP_Xe_ (%)	31.0 (12.1–47.1)	39.6 (23.6–51.1)	6.4 (2.8–12.4)	< 0.0001*

All data is presented as median with interquartile range in brackets

Differences in demographic, spirometric, and MRI-derived parameters between COPD and healthy controls were tested for significance by the Wilcoxon rank sum test Statistically significant differences (*p* < 0.05) are indicated with *

*BMI* body mass index, *COPD* chronic obstructive pulmonary disease, *FEV*_*1*_ forced expiratory volume in 1 s, *FVC* forced vital capacity, *GOLD* the global initiative for chronic obstructive lung disease, *VDP*_*Combined*_ ventilation defect percentage (VDP) derived by 3D PREFUL MRI using both regional ventilation (RVent) and flow-volume-loop correlation metric (FVL-CM), *VDP*_*FVL-CM*_ VDP derived by 3D PREFUL MRI using FVL-CM, *VDP*_*RVent*_ VDP derived by 3D PREFUL MRI using RVent, *VDP*_*Xe*_ VDP derived ^129^Xe MRI

### Global comparison of 3D PREFUL MRI with ^129^Xe MRI

**Fig. 1:**
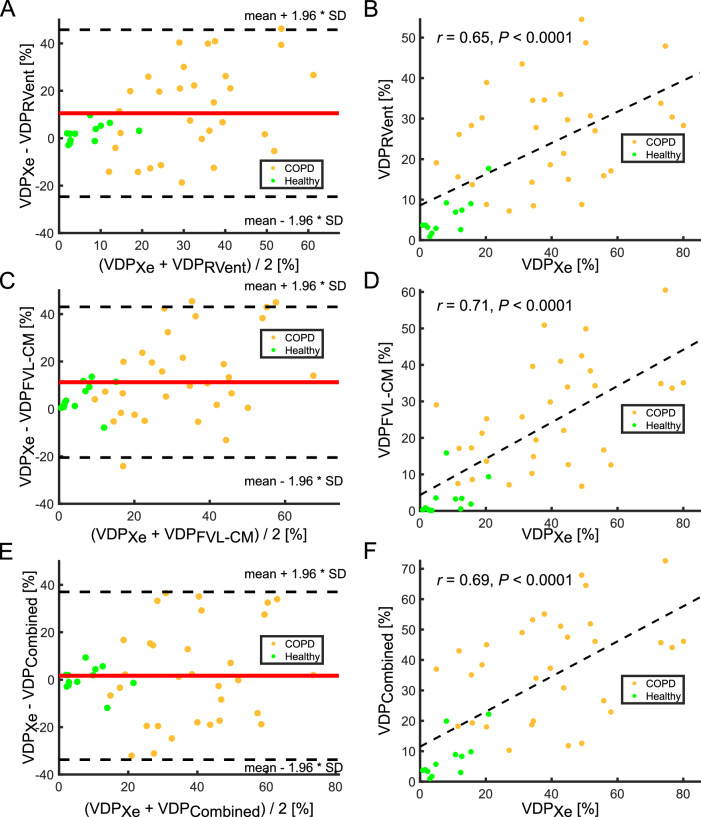
Bland–Altman analysis of VDP_RVent_ (**A**), VDP_FVL-CM_ (**C**), and VDP_Combined_ (**E**) values derived by 3D PREFUL MRI with VDP value derived by ^129^Xe MRI. The mean differences and limits of agreement (mean ± 1.96 *SD) are shown by solid red and dashed black lines, respectively. The corresponding regression analysis between 3D PREFUL VDP parameters and VDP derived by ^129^Xe MRI is depicted in **B** for VDP_RVent_; **D** for VDP_FVL-CM_; and in **F** for VDP_Combined_. Gold dots: COPD patients; green dots: healthy volunteers

Table 2 presents the correlation analysis (A) between 3D PREFUL MRI and ^129^Xe MRI. All 3D PREFUL MRI-derived VDP values were significantly correlated with ^129^Xe MRI-derived VDP value (all *r* ≥ 0.65, all *p* < 0.0001). For VDP_RVent_, Bland–Altman analysis revealed significant differences to ^129^Xe MRI VDP (mean bias = 10.5%, *p* = 0.0010, Table 2B and Fig. 1A). Similarly, a significant bias was found between VDP_FVL-CM_ and VDP_Xe_ (mean bias = 11.3%, *p* < 0.0001, Table 2B and Fig. 1D). Considering VDP_Combined_, no significant differences were observed in comparison to VDP_Xe_ (mean bias = 1.7%, *p* = 0.70, Table 2B and Fig. 1F).

**Table 2:** Spearman correlation (A) and Bland–Altman analysis (B) of 3D PREFUL MRI with ^129^Xe MRI ventilation imaging for all study participants

A. Correlation analysis	VDP_RVent_		VDP_FVL-CM_		VDP_Combined_	
	*r*	*p*	*r*	*p*	*r*	*p*
VDP_Xe_	0.65	< 0.0001*	0.71	< 0.0001*	0.69	< 0.0001*

B. Bland–Altman analysis	VDP_RVent_		VDP_FVL-CM_		VDP_Combined_	
	Mean bias (LoA)	*p*	Mean bias (LoA)	*p*	Mean bias (LoA)	*p*
VDP_Xe_	10.5 (−24.7 to 45.7)	0.0010*	11.3 (−20.5 to 43.0)	< 0.0001*	1.7 (−33.7 to 37.0)	0.70

In (A) VD percentage values were compared using Spearman correlation analysis (*r*). * Statistically significant correlations

In (B) VDP values derived by 3D PREFUL MRI and ^129^Xe MRI were compared using Bland–Altman analysis (presented as a mean bias with limits of agreement in brackets) and the differences were tested for significance by paired Wilcoxon signed rank test. * Statistically significant correlations and mean differences (*p* < 0.0167)

*LoA* limits of agreement, *VDP*_*Combined*_ ventilation defect percentage (VDP) derived by 3D PREFUL MRI using both RVent and flow-volume-loop correlation metric (FVL-CM), *VDP*_*FVL-CM*_ VDP derived by 3D PREFUL MRI using FVL-CM, *VDP*_*RVen*_*t* VDP derived by 3D PREFUL MRI using RVent, *VDP*_*Xe*_ VDP derived from ^129^Xe MRI

### Regional comparison of VDP values derived by 3D PREFUL MRI and ^129^Xe MRI

**Fig. 2:**
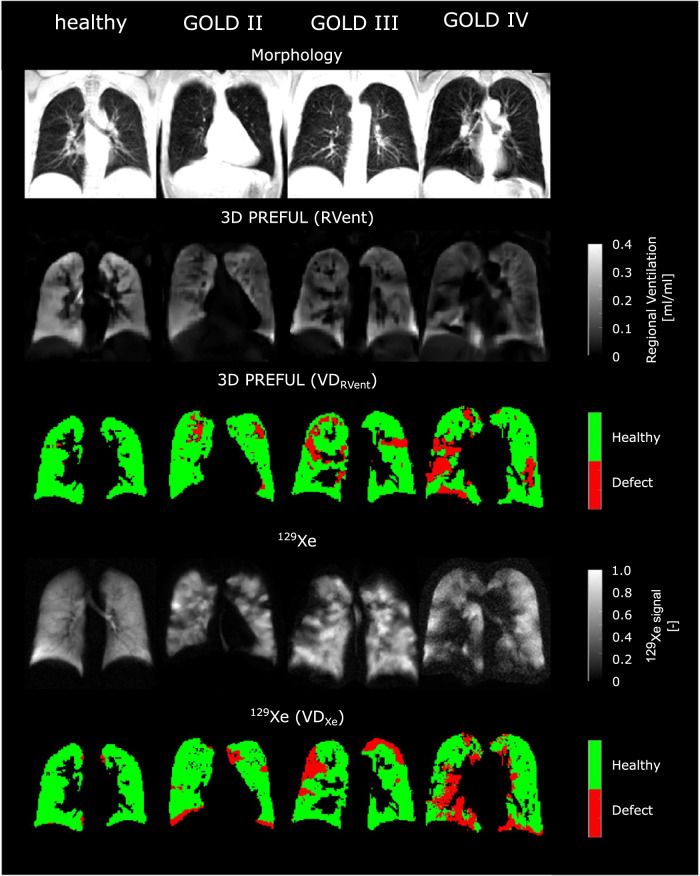
Representative morphological images (1st row) and ventilation parameter maps of four study participants derived by 3D PREFUL MRI (2nd and 3rd row, static RVent-based parameter maps) and ^129^Xe MRI (4th and 5th row). First column: a male 29-year-old healthy volunteer (FEV_1_ = 80% pred., FVC = 85% pred.); second column: a female 67-year-old COPD patient (FEV_1_ = 66% pred., FVC = 103% pred., GOLD II); third column: a male 60-year-old COPD patient (FEV_1_ = 39% pred., FVC = 66% pred., GOLD III); fourth column: a male 70-year-old COPD patient (FEV_1_ = 28% pred., FVC = 64% pred., GOLD IV)

**Fig. 3:**
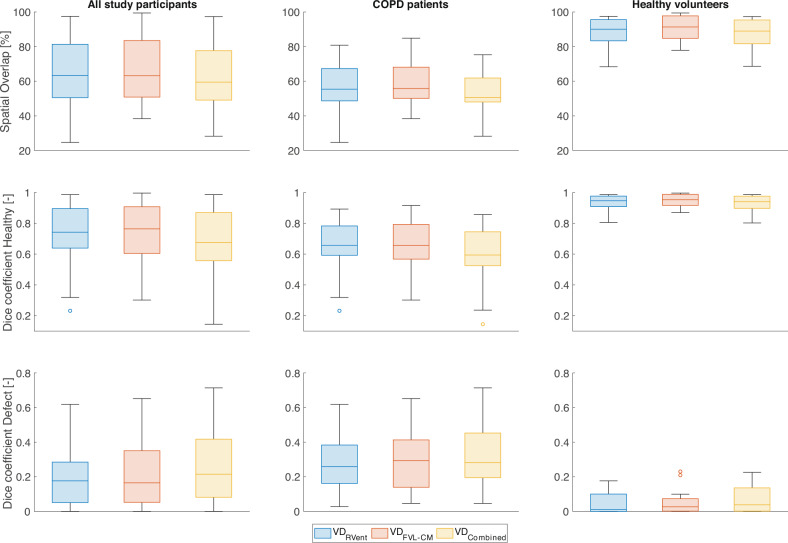
Regional agreement of VD maps derived by 3D PREFUL and ^129^Xe MRI. The overall spatial agreement of both methods was evaluated by spatial overlap metric (1st row) and Sørensen–Dice coefficients in healthy (2nd row) and defect voxels (3rd row). The analysis was conducted in all study participants (1st column), in COPD patients (2nd column), and in healthy volunteers (3rd column). Blue boxes = VD_RVent_; red boxes = VD_FVL-CM_; yellow boxes = VD_Combined_

**Fig. 4:**
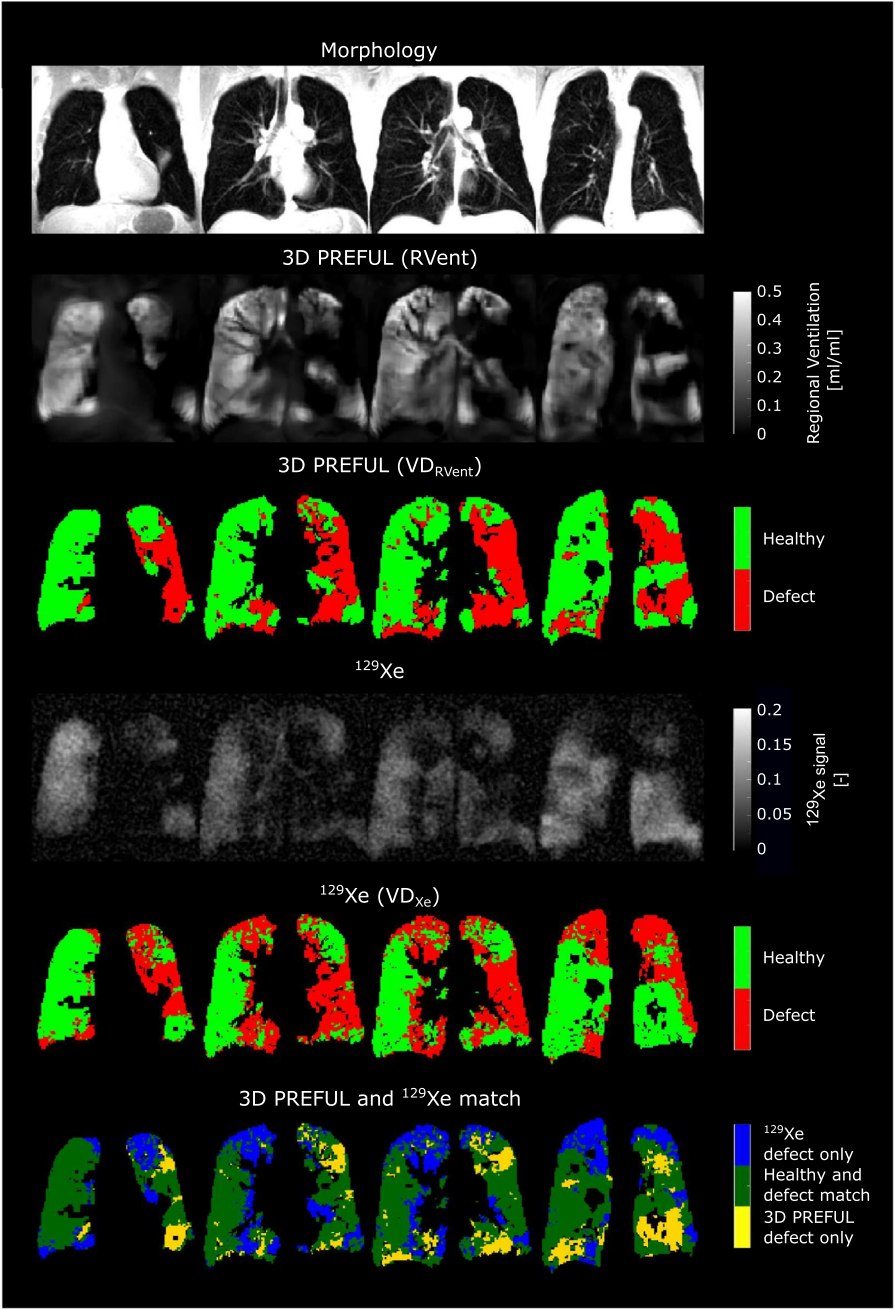
Exemplary morphological images (1st row) and respective ventilation parameter maps of a COPD patient (68-year-old male, FEV_1_ = 22% pred., FVC = 74% pred., GOLD IV) by 3D PREFUL MRI (2nd and 3rd row, static RVent based parameter maps) and ^129^Xe MRI (4th and 5th row), as well as spatial agreement of VD maps of both techniques (6th row). In all evaluated slices, the Sørensen–Dice coefficients were 0.52 in defect and 0.75 in healthy areas, which resulted in a total spatial overlap of 66.8%. Note the matched VDs and healthy areas (dark green) in both methods, along with minor differences in blue and yellow

**Fig. 5:**
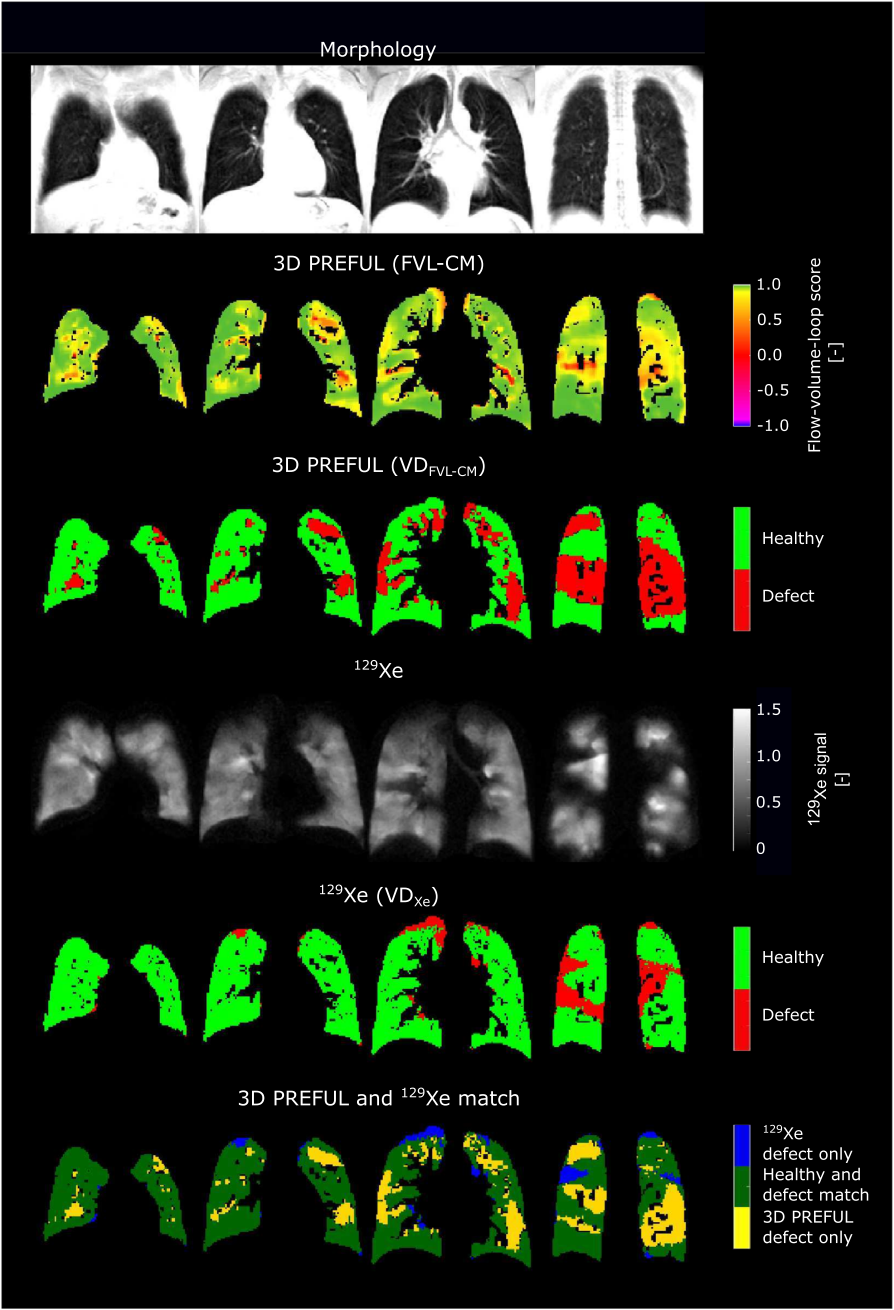
Exemplary morphological images (1st row) and respective ventilation parameter maps of a COPD patient (60-year-old female, FEV_1_ = 58% pred., FVC = 77% pred., GOLD II) by 3D PREFUL MRI (2nd and 3rd row, dynamic flow-volume loop based parameter maps) and ^129^Xe MRI (4th and 5th row), as well as spatial agreement of VD maps of both techniques (6th row). In all evaluated slices, the Sørensen–Dice coefficients were 0.29 in defect and 0.89 in healthy areas, which resulted in a total spatial overlap of 81.8%. Note the matched VDs and healthy areas (dark green) in both methods, along with minor differences in blue and yellow

**Fig. 6:**
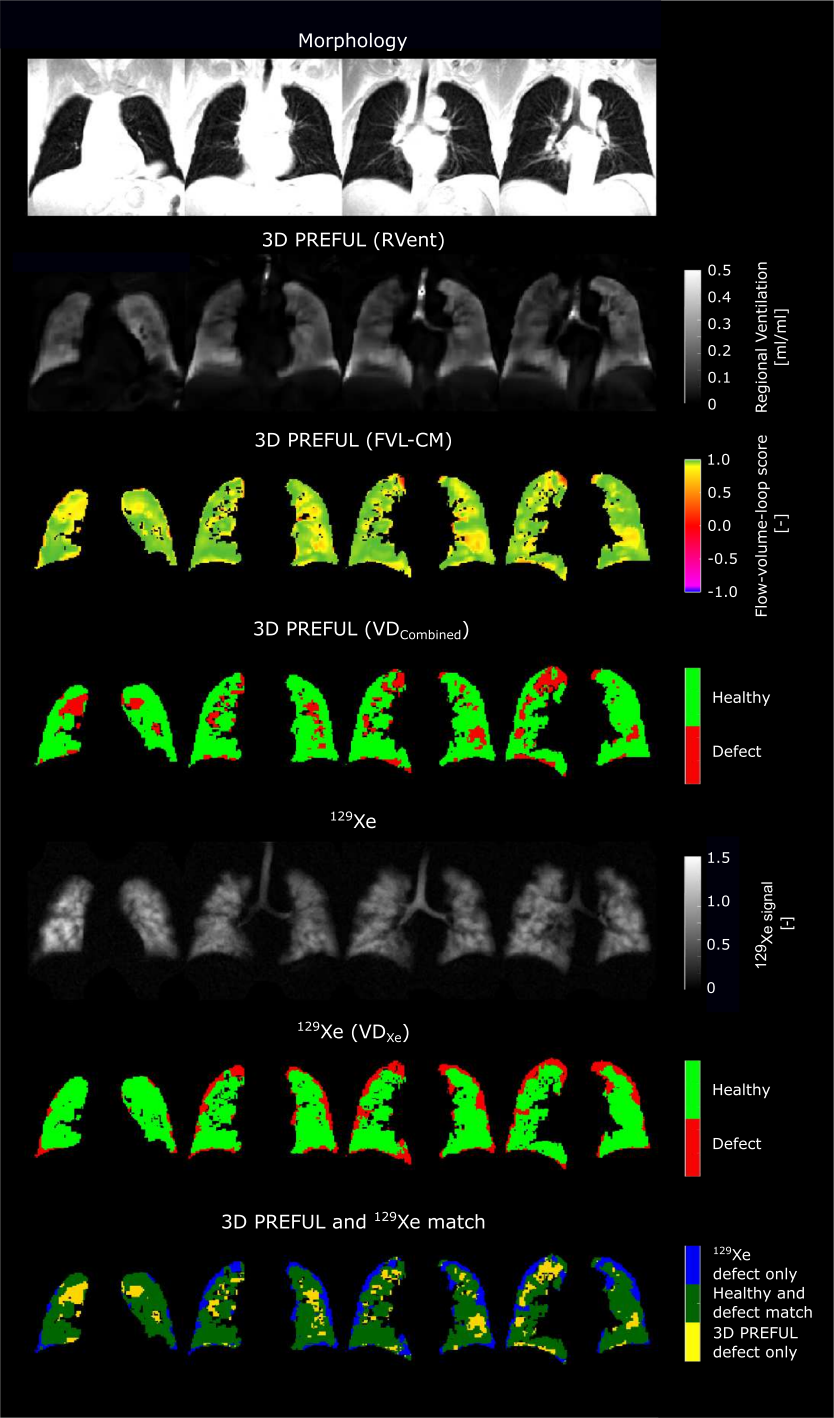
Exemplary morphological images (1st row) and respective ventilation parameter maps of a COPD patient (64-year-old male, FEV_1_ = 35% pred., FVC = 88% pred., GOLD III) by 3D PREFUL MRI (2nd–4th row, static RVent and dynamic flow-volume loop based parameter maps and combination of them) and ^129^Xe MRI (5th and 6th row), as well as spatial agreement of VD maps of both techniques (7th row). In all evaluated slices, the Sørensen–Dice coefficients were 0.17 in defect and 0.83 in healthy areas, which resulted in a total spatial overlap of 72.4%. Note the matched VDs and healthy areas (dark green) in both methods, along with minor differences in blue and yellow

Figure 2 and Supporting Fig. 2 show representative examples of ventilation parameters derived by 3D PREFUL and ^129^Xe MRI for COPD patients and a healthy study participant, with good visual-spatial correspondence. The total spatial agreement between 3D PREFUL MRI VD maps and ^129^Xe MRI VD maps ranged in the median from 60% up to 63% in the whole study population. Sørensen–Dice coefficients evaluated in healthy regions of all study participants ranged between 0.67 and 0.76, with the highest coefficients for FVL-CM derived maps. For the corresponding defect regions of the whole study cohort, the Sørensen–Dice coefficients were in the median between 0.16 and 0.22, with the highest values for the combination of RVent and FVL-CM parameters. The identical regional analysis for both COPD patients and healthy volunteers is presented in Fig. 3 and Supporting Table S2. Representative ventilation marker maps of both techniques (including their spatial correspondence) for COPD patients are shown in Figs. 4–6.

### Correlations of VDP values derived by 3D PREFUL and ^129^Xe MRI with spirometry

Table 3 presents the correlations between 3D PREFUL MRI, ^129^Xe MRI, and pulmonary functional testing parameters. For 3D PREFUL MRI, VDP_FVL-CM_ showed the strongest correlations to FEV_1_ and FEV_1_/FVC ratio (*r* ≥ −0.78, *p* < 0.0001). The VDP values derived by ^129^Xe MRI showed a similarly strong correlation to FEV_1_ and FEV_1_/FVC ratio (*r* ≥ −0.71, *p* < 0.0001).

**Table 3:** Correlation of 3D PREFUL MRI and ^129^Xe MRI ventilation parameters with spirometry outcomes

Ventilation parameter	FEV_1_, (% pred.)		FVC, (% pred.)		FEV_1_/FVC, (% pred.)	
	*r*	*p*	*r*	*p*	*r*	*p*
VDP_RVent_	−0.74	< 0.0001*	−0.37	0.0135	−0.71	< 0.0001*
VDP_FVL-CM_	−0.81	< 0.0001*	−0.43	0.0043*	−0.78	< 0.0001*
VDP_Combined_	−0.77	< 0.0001*	−0.45	0.0028*	−0.73	< 0.0001*
VDP_Xe_	−0.71	< 0.0001*	−0.23	0.14	−0.74	< 0.0001*

MRI ventilation parameters were compared to spirometry outcomes using Spearman correlation analysis (*r*), statistically significant correlations (with Bonferroni corrected *p* < 0.125) are indicated with *

## Discussion

This study presents a direct comparison of 3D PREFUL MRI to ^129^Xe MRI in patients with COPD and healthy volunteers. The main results are: (1) global VD percentage values of 3D PREFUL MRI are significantly correlated with VD percentage values of hyperpolarized ^129^Xe MRI; (2) ^129^Xe MRI VDP values were significantly greater than VDP values of 3D PREFUL; (3) regional voxelwise assessment of binary VD maps showed spatial overlap up to 63%; and (4) both methods correlated well with standard ventilation measures derived by pulmonary function testing.

In all study participants, a strong global VDP correlation was observed between 3D PREFUL MRI and ^129^Xe MRI. The dynamic FVL-CM parameter of 3D PREFUL VDP showed the strongest positive relationship with ^129^Xe MRI VDP, consistent with previous studies [17, 26], in patients with cystic fibrosis and COPD. However, a significant bias of approximately 10% lower VDP values (VDP_RVent_ and VDP_FVL-CM_) was noted for 3D PREFUL compared to ^129^Xe MRI, which increased with disease severity. Although the largest bias was seen for VDP_FVL-CM_, the narrowest agreement levels were found for this parameter compared to VDP_Xe_. The inter-technique bias and broad levels of agreement of VDP values can be attributed to several explanations. First, the principles of creating and capturing the ventilation signal differ. In the case of 3D PREFUL, it relies on an indirect approach, where the ventilation signal is derived under the assumption that the MR signal within lung parenchyma is proportional to lung volume, which varies during a free-breathing acquisition [13]. For ^129^Xe imaging, a hyperpolarized high-contrast gas, in combination with nitrogen, is inhaled from the Tedlar bag. A snapshot breath-hold image is acquired using dedicated coils for ^129^Xe imaging while the lungs are filled with inhaled gas. Secondly, the spatial resolution of both methods slightly differed regarding the slice thickness: 3.9 mm for 3D PREFUL MRI as opposed to 15 mm for ^129^Xe MRI. Thirdly, in slow-filling areas of the lungs of COPD patients, the inhaled gas mixture may not have fully reached those lung areas. These lung regions might be depicted as VDs in ^129^Xe MRI, even though they may still be filled through collateral ventilation [40]. Considering the flow-volume-loop derived VDP (VDP_FVL-CM_) of 3D PREFUL, this marker showed a stronger correlation with the VDP values derived by ^129^Xe MRI and is likely more sensitive to subtle changes. Fourth, different threshold techniques for VD calculation may also contribute to discrepancies, though this study did not aim to determine optimal thresholds but rather assess overall and regional correspondence using established thresholds.

Contrary to previously mentioned results, for the VDP values which combined both 3D PREFUL parameters (RVent and FVL-CM) no significant bias was found to ^129^Xe MRI VDP. In previous work, a combination of VDP_RVent_ and VDP_FVL-CM_ has been shown to improve the correlation of 2D PREFUL MRI to ^129^Xe MRI [17]. In our study, the correlation between global VDP_Combined_ values and VDP values from ^129^Xe MRI remained comparable to the correlation observed for VDP_RVent_ and VDP_FVL-CM_. This finding is consistent with the work of Munidasa et al [11], who also did not observe any increase in correlation between VDP_Combined_ derived by 2D PREFUL MRI and VDP value derived by hyperpolarized ^129^Xe gas MRI in cystic fibrosis and healthy volunteers cohort.

In most study participants, a spatial concordance between VD maps derived by 3D PREFUL MRI and ^129^Xe MRI was found. However, certain regions exhibited disagreement, with mislabelling of both healthy and defective areas observed in up to 40% of regions within the 3D PREFUL maps, visible as blue and yellow areas in Figs. 5 and 6. Combining VDs from RVent and FVL-CM maps of 3D PREFUL MRI accurately depicted VDs identified by ^129^Xe MRI, as seen in previous validation studies [11, 17]. This underscores the complementary nature of both markers in capturing ventilation abnormalities. The lack of absolute agreement in VD detection was not unexpected and might be explained by several factors. Unlike hyperpolarized gas ^129^Xe MRI, 3D PREFUL assesses ventilation dynamics during free-breathing, where varying lung volumes can lead to different VDP values [41]. Consequently, some of the discrepancies in VD maps might be explained by the different lung volumes between both approaches. Further, 3D PREFUL relies on image registration which corrects for the lung and cardiac motion, potentially introducing minor errors near the diaphragm, heart, or lung cavity borders. Since the ^129^Xe images are acquired during breath-hold, the measurements are dependent on subject compliance and motivation to follow the breathing instructions. Not following the instructions correctly results in inaccurate VD calculation, particularly near the diaphragm. Enhancements to the 3D PREFUL pipeline can be realized through improvements in image reconstruction, particularly with recent advancements like MoCoLor [42]. This technique integrates motion compensation directly into a low-rank constrained reconstruction model, optimizing the utilization of acquired data for functional proton MRI. Furthermore, the incorporation of additional ventilation markers such as PREFUL parametric response mapping (PRM) [43] has demonstrated the ability to detect abnormalities potentially missed by the conventional regional ventilation (RVent) approach. These advancements show great potential for refining assumptions and bolstering the reliability of 3D PREFUL methodology.

Finally, VDP values quantified by 3D PREFUL MRI and ^129^Xe MRI were comparably well correlated to standard measures of pulmonary functional testing. This finding is consistent with previous studies [17, 24, 26]. The current management of COPD patients relies solely on spirometry. However, assessing this highly complex disease based on global spirometry values might not be sufficiently accurate and is not feasible in some cases. 3D PREFUL MRI might potentially serve as a valuable additional tool for the evaluation of disease progression and may even substitute spirometry in certain situations, particularly in patient monitoring.

This work was limited by the definitions of thresholds, which were used for the VD/VDP calculation of both methods. The 3D PREFUL approach utilizes both variable (RVent) and fixed (FVL-CM) thresholds. In ^129^Xe imaging, a linear binning method [38] was employed. The use of other non-established thresholds would yield different results in global and regional analysis.

Compared to the current gold standard ventilation imaging of SPECT, hyperpolarized gas MRI provides improved spatial and temporal resolution without the use of ionizing radiation. Previous cross-modality studies reported moderate to good agreement on global/lobar level (*r* range: 0.48–0.64) between ventilation SPECT using technetium-99m-labeled aerosol and ^129^Xe MR imaging [44, 45]. Compared to previous results, our analysis showed positive correlations with superior values (*r* range: 0.65–0.71), suggesting for strong agreement of 3D PREFUL MRI with ^129^Xe imaging on the global level. The objective of a subsequent study might involve a direct comparison of both MR techniques with the clinical standard measurement of SPECT imaging.

Our study participants consisted of COPD patients and healthy volunteers, who were not age- and sex-matched. Consequently, no reasonable comparison was possible between both cohorts in this setting. Additional research is needed to validate the 3D PREFUL MRI technique in other patient populations and within a multicenter setting. Moreover, future work is also required to determine whether both measurements yield comparable responsiveness to treatment.

## Conclusions

Ventilation markers obtained through 3D PREFUL MRI exhibited a strong global correlation with ^129^Xe MRI, revealing modest regional variations in identifying VDs in patients with COPD. These findings suggest that 3D PREFUL ventilation MRI may offer surrogate markers for ventilation and could potentially provide an alternative to ^129^Xe MRI ventilation mapping. Notably, 3D PREFUL avoids the necessity for costly additional MRI hardware and the administration of inhaled gases.

## Supplementary information

Electronic Supplementary Material

## Abbreviations

BMI: Body mass index
COPD: Chronic obstructive lung disease
CT: Computed tomography
FEV_1_: Forced expiratory volume in 1 s
FVC: Forced vital capacity
FVL-CM: Flow-volume-loop cross-correlation metric
MRI: Magnetic resonance imaging
PREFUL: Phase-resolved functional lung
RVent: Regional ventilation
SPECT: Single photon emission computed tomography
VD: Ventilation defect
VDP: Ventilation defect percentage (%)
