# Supplementary material for: Free-breathing 3D phase-resolved functional lung MRI vs breath-hold hyperpolarized 129Xe ventilation MRI in patients with chronic obstructive pulmonary disease and healthy volunteers
Source: Eur Radiol. 2024 Jul 26;35(2):943–56. doi: 10.1007/s00330-024-10893-3 (PMC11782336; doi:10.1007/s00330-024-10893-3)
Supplement: Supplementary file 1 — Electronic Supplementary Material [file 330_2024_10893_MOESM1_ESM.pdf]

Free-breathing 3D phase-resolved functional lung MRI versus ^{129}Xe ventilation MRI in patients with chronic obstructive pulmonary disease

Electronic Supplementary Material (ESM)

Eur Radiol (2024) Klimeš F, Kern AL, Voskrebenzev A et al.

Supporting Information

<u>3D PREFUL MRI image reconstruction</u>

After the MR acquisition with stack of stars trajectory, the obtained radial spokes were sorted into bins based according to their corresponding respiratory phase. The respiratory phase of each projection was determined from the lung parenchyma segmentation of the 3D low-resolution images. The low-resolution images with temporal resolution of approximately 100 ms and spatial resolution of 15.6 mm^3 were reconstructed using the parallel imaging and compressed sensing (PICS) algorithm implemented in the Berkeley Advanced Reconstruction Toolbox (BART), so that for each low-resolution image 14 neighboring spokes were considered for the reconstruction. The lung parenchyma of each low-resolution image was segmented using Otsu's thresholding and the lung volume was computed. The total lung volume was then used as a binning signal. The reconstruction of full resolution images with spatial resolution of 3.9 mm x 3.9 mm x 3.9 mm was accomplished as follows:

1) Each respiratory bin contained a fixed minimum of 100 spokes, which gave us approximately 40 respiratory phases for each study participant.
2) Additionally, for each respiratory bin, we considered the peripheral part of spokes, specifically limited to the initial and final quarters of each radial spoke within the two neighboring groups. This approach aims to enhance the signal-to-noise ratio (SNR).
3) To reconstruct temporally resolved full resolution images, the PICS algorithm implemented in BART software, was utilized. The sparsity was endorsed in both spatial and time domains through the use of the L2 norm and total variation for the spatial domain, and total variation for the temporal dimension.

Next, a group-oriented image registration of the N4 bias field-corrected full resolution images towards end-inspiratory state, using the Forsberg registration toolbox was applied.

Subsequently, a Gaussian kernel was employed to interpolate 16 respiratory phases on an equidistant time grid. Following the removal of signal modulations unrelated to respiration, which was executed through a low-pass filtering with a cutoff frequency at 0.7 Hz, a 3D edge-preserving filter was applied to the low-pass filtered images.

Supporting Information Figures

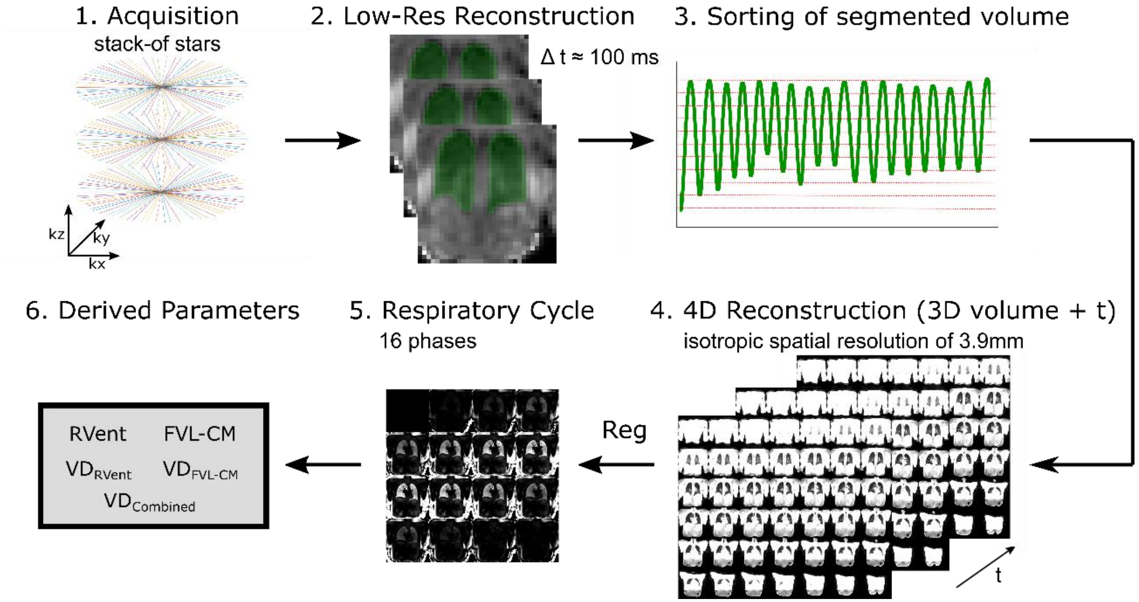

Supporting Fig. 1 Schematic overview of 3D PREFUL method. (1) Data are acquired using a prototype stack-of-stars acquisition for approximately 8 minutes. (2) Low-resolution images, with a matrix size of 32 × 32 × 12-18 and a temporal resolution of approximately 100 ms, are reconstructed. (3) A gating signal is derived from a segmentation of lung parenchyma and is used to sort acquired spokes into respiratory bins. (4) Dynamic images in full spatial resolution (interpolated to 3.9 × 3.9 × 3.9 mm³) are reconstructed. (5) After registration towards a reference max-inspiratory image, the images are interpolated to a uniform time grid, and a respiratory cycle comprising 16 phases is computed. (6) Finally, ventilation parameters are obtained: FVL-CM (flow-volume-loop correlation metric) map, RVent (regional ventilation) map, $VD_{FVL\text{-}CM}$

(ventilation defect map based on FVL-CM), VD_{RVent} (ventilation defect map based on RVent), and $VD_{Combined}$ (ventilation defect map combining FVL-CM and RVent).

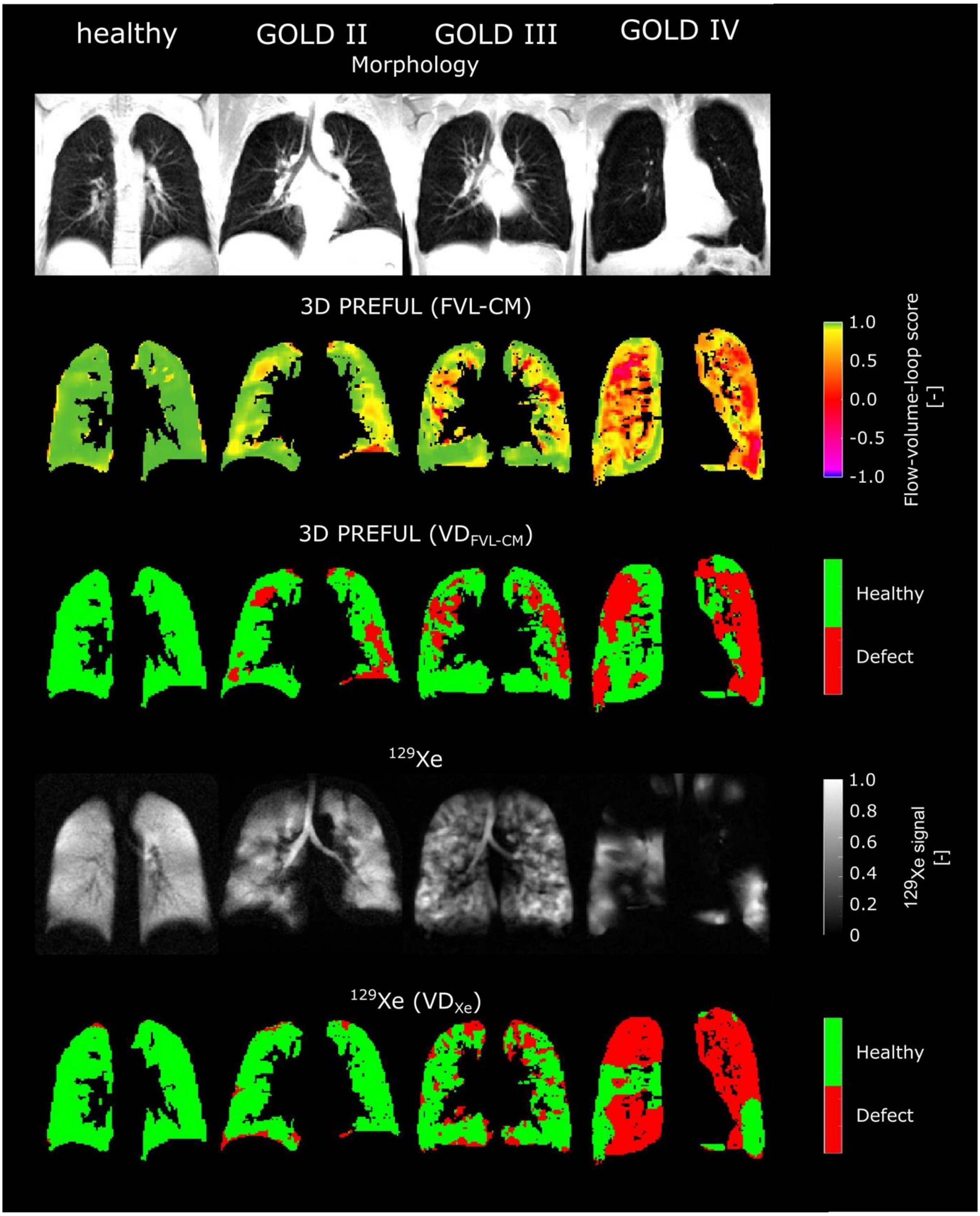

Supporting Fig. 2 Representative morphological images (1st row) and ventilation parameter maps of four study participants derived by 3D PREFUL MRI (2nd and 3rd row, dynamic flow-volume loop based parameter maps) and ^{129}Xe MRI (4th and 5th row). First column: a male 25-year-old healthy volunteer (FEV_1 = 102% pred., FVC = 116% pred.); second column: a male 67-year-old COPD patient (FEV_1 = 78% pred., FVC = 94% pred., GOLD II); third column: a

female 42-year-old COPD patient (FEV_1 = 35% pred., FVC = 88% pred., GOLD III); fourth column: a male 68-year-old COPD patient (FEV_1 = 20% pred., FVC = 50% pred., GOLD IV).

Supporting Information Tables

Supporting Table S1 MRI sequence parameters for 3D PREFUL MRI and ^{129}Xe MRI

Parameter / Method	3D PREFUL MRI	^{129}Xe MRI
Pulse sequence type	3D spoiled gradient echo	3D balanced steady state free precession
Trajectory	Stack-of-stars	Stack-of-stars
FOV	500 x 500 x (203-313)	350 x 350 x 240
Imaging Matrix	128 x 128 x (52-80)	128 x 128 x 16
Slice thickness (mm)	3.9	15
TR (ms)	1.9	3.69
TE (ms)	0.81	1.78
Flip angle (°)	3.5	13
Bandwidth (Hz/pixel)	1500	890
Acquisition Time (s)	436 - 498	5
Slice partial Fourier	6/8	8/8

FOV = field of view; TE = echo time; TR = repetition time.

Supporting Table S2 Regional agreement of 3D PREFUL MRI and ^{129}Xe MRI derived ventilation defect (VD) maps for all study participants (A), for COPD patients only (B) and for healthy controls only (C).

**A.**	**VD_{Xe}**		
	**Spatial overlap (%)**	**Dice Healthy (-)**	**Dice Defect (-)**
**VD_{RVent}**	63.3 (50.6-81.1)	0.74 (0.64-0.89)	0.18 (0.05-0.28)
**VD_{FVL-CM}**	63.2 (50.8-82.9)	0.76 (0.61-0.90)	0.16 (0.05-0.35)
**$VD_{Combined}$**	59.5 (49.2-76.9)	0.67 (0.56-0.87)	0.22 (0.08-0.41)
**B.**	**Spatial overlap (%)**	**Dice Healthy (-)**	**Dice Defect (-)**
**VD_{RVent}**	55.4 (48.7-67.2)	0.66 (0.60-0.77)	0.26 (0.16-0.38)
**VD_{FVL-CM}**	55.8 (50.1-67.4)	0.66 (0.57-0.79)	0.29 (0.14-0.40)
**$VD_{Combined}$**	50.5 (48.2-61.6)	0.59 (0.53-0.74)	0.28 (0.20-0.45)
**C.**	**Spatial overlap (%)**	**Dice Healthy (-)**	**Dice Defect (-)**
**VD_{RVent}**	90.0 (83.5-95.5)	0.95 (0.91-0.98)	0.01 (0.00-0.08)
**VD_{FVL-CM}**	91.3 (85.1-97.6)	0.95 (0.92-0.99)	0.03 (0.00-0.06)
**$VD_{Combined}$**	88.9 (81.8-95.4)	0.94 (0.90-0.98)	0.04 (0.00-0.11)

All data is presented as median with interquartile range in brackets.

$VD_{Combined}$, ventilation defect (VD) maps derived by 3D PREFUL MRI using both regional ventilation (RVent) and flow-volume-loop correlation metric (FVL-CM); VD_{FVL-CM}, VD maps derived by 3D PREFUL MRI using FVL-CM; VD_{RVent}, VD maps derived by 3D PREFUL MRI using RVent; VDP_{Xe}, VD maps derived ^{129}Xe MRI.
